# P14 A case of myositis in patient with atypical bacterial pneumonia

**DOI:** 10.1093/rap/rkab068.013

**Published:** 2021-10-19

**Authors:** Smrity Smrity, Cathy Lawson, Sarah Marsh

**Affiliations:** Harrogate District Hospital, Harrogate, United Kingdom

## Abstract

**Case report - Introduction:**

Bacterial community-acquired atypical pneumonia is sometimes complicated by myositis or by renal parenchymal disease. They can present with myositis and present with muscle weakness, pain or swelling, and elevated muscle enzymes. We present the case of a patient with lower limb weakness and raised creatinine kinase with atypical pneumonia caused by *Legionella pneumophila*.

**Case report - Case description:**

A 76-year-old Caucasian man, who was previously fit and independent and walked 3 miles every day presented with a 1-week history of progressive leg weakness, and inability to mobilize. He had a fall and was on the floor for 2 hours. He had a background history of hypercholesterolemia and was on atorvastatin for 15 years. On his vital observation, he was found tachypnoeic, tachycardic, and hypoxic. He had a right upper lobe crackle but he didn’t have respiratory symptoms. His muscle power in his leg was 3/5 with carpet burns on knees and elbow.

Initial investigation showed raised inflammatory marker CRP 412mg/L, AKI stage 1, and CK 43400 IU/L. His CXR showed dense right upper lobe consolidation. Legionella urinary antigen was positive. Myositis myoblot, ANA, ANCA negative. COVID-19 swab negative.

Treated with IV antibiotic, supplemental oxygen, and IV fluid. Transferred to ITU due to worsening of hypoxia and kidney function. Interestingly, the CK level had improved significantly within 48 hours along with clinical improvement in his symptoms. There was no role of steroid or immunosuppressant due to his significant clinical improvement. On day 7 he was off oxygen, kidney function improved, had physiotherapy, and transferred to ward and on day 10 he was ambulant and discharged home.

**Case report - Discussion:**

To date, very few case reports of myositis in a patient with atypical pneumonia have been reported. The mechanism underlying acute myositis in atypical pneumonia is still unknown. The present analysis points out that the organism underlying atypical bacterial pneumonia may occasionally invade the muscle tissue thereby inducing both myositis and secondary kidney damage.

**Case report - Key learning points:**

We should be aware of this rare complication of atypical pneumonia and the resolution of symptoms that occur with the treatment of pneumonia. This would avoid unnecessary investigation and use of steroid.

